# Excess pneumonia and influenza mortality attributable to seasonal influenza in subtropical Shanghai, China

**DOI:** 10.1186/s12879-017-2863-1

**Published:** 2017-12-07

**Authors:** Xinchun Yu, Chunfang Wang, Tao Chen, Wenyi Zhang, Huiting Yu, Yuelong Shu, Wenbiao Hu, Xiling Wang

**Affiliations:** 1 Department of Biostatistics, School of Public Health, Fudan University, Key Laboratory of Public Health Safety, Ministry of Education, 200231 Xuhui District, Shanghai, China; 2 Shanghai Municipal Center for Disease Control and Prevention, Shanghai, China; 3 National Institute for Viral Disease Control and Prevention, China Centers for Disease Control and Prevention, Key Laboratory for Medical Virology, National Health and Family Planning Commission, Beijing, China; 4 Institute of Disease Control and Prevention, Academy of Military Medical Science, Beijing, China; 5 School of Public Health, Sun Yat-sen University, Shenzhen, China; 6 School of Public Health and Social Work, Queensland University of Technology, Kelvin Grove, Brisbane, QLD 4059 Australia; 7 Institute of Health and Biomedical Innovation, Queensland University of Technology, Kelvin Grove, Brisbane, QLD 4059 Australia; 8 Shanghai Key Laboratory of Meteorology and Health, Shanghai, China

**Keywords:** Influenza, Excess mortality, Quasi-Poisson modelm, Influenza-like illness

## Abstract

**Background:**

Disease burden attributable to influenza is substantial in subtropical regions. Our study aims to estimate excess pneumonia and influenza (P&I) mortality associated with influenza by subtypes/lineages in Shanghai, China, 2010–2015.

**Methods:**

Quasi-Poisson regression models were fitted to weekly numbers of deaths from causes coded as P&I for Shanghai general and registered population. Three proxies for influenza activity were respectively used as an explanatory variable. Long-term trend, seasonal trend and absolute humidity were adjusted for as confounding factors. The outcome measurements of excess P&I mortality associated with influenza subtypes/lineages were derived by subtracting the baseline mortality from fitted mortality.

**Results:**

Excess P&I mortality associated with influenza were 0.22, 0.30, and 0.23 per 100,000 population for three different proxies in Shanghai general population, lower than those in registered population (0.34, 0.48, and 0.36 per 100,000 population). Influenza B (Victoria) lineage did not contribute to excess P&I mortality (*P* = 0.206) while influenza B (Yamagata) lineage did (*P =* 0.044). Influenza-associated P&I mortality was high in the elderly population.

**Conclusions:**

Seasonal influenza A virus had a higher P&I mortality than influenza B virus, while B (Yamagata) lineage is the dominant lineage attributable to P&I mortality.

**Electronic supplementary material:**

The online version of this article (10.1186/s12879-017-2863-1) contains supplementary material, which is available to authorized users.

## Background

Seasonal influenza viruses circulate worldwide and cause severe illnesses and deaths every year [1, 2]. According to the World Health Organization (WHO), influenza can cause 3–5 million severe illnesses and 250,000 to 500,000 excess deaths every year [3]. In China, influenza cause hundreds of thousands of excess deaths every year [4]. Reliable quantification of disease burden of influenza is of key importance for policy-makers to properly allocate scarce medical resources.

However, quantification of disease burden of influenza remains challenging since it is difficult to differentiate influenza from illnesses caused by other respiratory pathogens based on non-specific clinical manifestations [5, 6]. Besides, current laboratory approaches have demonstrated limited capacity [7–9] and older people are likely to die from influenza-related complications [10], which may both lead to under-reporting. Statistical modeling provides an alternative in estimating disease burden of influenza [11], among which times series models have been widely used [12–14]. Quasi-Poisson regression modeling has been widely used to provide estimates of influenza disease burden by incorporating influenza surveillance data. The rationale of Quasi-Poisson model is based on influenza-associated excess mortality, defined as the difference between fitted number and baseline number by setting influenza proxy variable(s) to zero [8].

Different research groups have used different influenza proxies developed from influenza surveillance data to estimate influenza-associated disease burden, while different influenza proxies might lead to different estimates for excess mortality [15]. In addition, although great disease burden of influenza was revealed in the subtropical regions of China [16], the disease burden associated with influenza B by lineages has not been well explored. The “floating population”, a group of migrants without local household registration status (*hukou*), has attracted growing attention in China [17]. It has been reported that the floating population and the general population were at different risks for some infectious diseases and might have different health behaviors due to social and economic factors [18, 19]. However, the gap in influenza-associated mortality between floating population and registered residents has not been assessed.

In this study, we chose three commonly used proxy variables (the weekly positive numbers/proportions of laboratory-confirmed influenza, and the product of weekly proportion and influenza-like illness consultation rate (LAB × ILI)) to estimate excess pneumonia and influenza (P&I) mortality attributable to influenza A subtypes and B lineages in the general and registered population in Shanghai, China from 2010 to 2015.

## Methods

### Data

Shanghai is in East China and has a subtropical climate with four distinct seasons. Weekly numbers of deaths with causes of death coded as P&I (International Classification of Diseases, Tenth Revision (ICD10) J09-J18) [20] from 2010 to 2015 were retrieved from Shanghai Center for Disease Control and Prevention (SCDC). Deaths in the general population of Shanghai and registered Shanghai residents were included. The annual population data were obtained from the household registration department of Shanghai Municipal Bureau of Public Security (http://www.stats.gov.cn/english/Statisticaldata/AnnualData/). The residing population of Shanghai can be sub-classified into two main groups, the Shanghai registered residents who permanently reside in Shanghai and have *hukou* (registration status), and those who might live for several months or years, i.e. non-registered temporary migrants with an alias as the “floating population” [21].

Influenza surveillance data were collected from National Influenza Center, China Center for Disease Control and Prevention (China CDC), including weekly numbers of specimens tested positive for influenza A(H1N1), influenza A(H3N2), influenza B (Victoria) lineage and influenza B (Yamagata) lineage, as well as the numbers of weekly specimens tested in total. Clinical ILI surveillance data consisted of weekly total outpatient visits and weekly numbers of ILI consultations. The definition of ILI was body temperature ≥ 38 *°C* with either sore throat or cough, without an alternative diagnosis [8]. The influenza surveillance protocol in Shanghai was in accord with the national influenza surveillance protocol and has been stated elsewhere [22].

Meteorological data for the years 2010–2015, including temperature and dew point temperature, were obtained from the China Meteorological Administration (CMA), which was an open-access website providing nationwide meteorological data (http://www.cma.gov.cn/en2014/). We aggregated hourly data into weekly mean temperature and weekly mean dew point temperature. We derived weekly absolute humidity by combing temperature and dewpoint temperature. Monday was regarded as the beginning of a week [23]. This study used aggregated data without personal information, so did not require ethical approval.

### Statistical analysis

The sampling of respiratory specimens was gradually enhanced, with the total specimens tested in 2015 nearly doubled the number in 2011. To reduce the impact of changing sampling behavior, our study considered three different proxies for influenza activity, including weekly positive numbers/proportions of lab-confirmed influenza, and LAB × ILI. These proxies represented the activity of influenza viruses [15]. Generalized additive models with the three proxies were used to fit weekly deaths coded as P&I in Shanghai general and registered population from 2010 to 2015, respectively. We adjusted for the confounding factors of long-term trend and seasonal trend. We additionally adjusted for absolute humidity in our model, as both laboratory and epidemiological studies have indicated that absolute humidity modulates influenza virus survival, seasonality and transmission [24]. The full model is as follows:

$$ \left\{\begin{array}{l}{Y}_t\sim quasiPoisson\left({\mu}_t,{\varphi \mu}_t\right)\\ {}\mathit{\log}\left({\mu}_t\right)={\beta}_0+{\beta}_1{(H1)}_{t-i}+{\beta}_2{(H3)}_{t-i}+{\beta}_3{(BV)}_{t-i}\ \\ {}\kern1.5em +{\beta}_4{(BY)}_{t-i}+s\left(t, df=3\times 6\right)+s\left({AH}_t, df=3\right)\ \end{array}\right. $$

$$ \left\{\begin{array}{l}{Y}_t\sim quasiPoisson\left({\mu}_t,{\varphi \mu}_t\right)\\ {}\mathit{\log}\left({\mu}_t\right)={\beta}_0+{\beta}_1{\left({LAB}_{H1}\right)}_{t-i}+{\beta}_2{\left({LAB}_{H3}\right)}_{t-i}+{\beta}_3{\left({LAB}_{BV}\right)}_{t-i}\\ {}\kern4.5em +{\beta}_4{\left({LAB}_{BY}\right)}_{t-i}+s\left(t, df=3\times 6\right)+s\left({AH}_t, df=3\right)\end{array}\right. $$

$$ \left\{\begin{array}{l}\kern1em {Y}_t\sim quasiPoisson\left({\mu}_t,{\varphi \mu}_t\right)\\ {}\mathit{\log}\left({\mu}_t\right)={\beta}_0+{\beta}_1{\left({LAB}_{H1}\times ILI\right)}_{t-i}+{\beta}_2{\left({LAB}_{H3}\times ILI\right)}_{t-i}+{\beta}_3{\left({LAB}_{BV}\times ILI\right)}_{t-i}\\ {}+{\beta}_4{\left({LAB}_{BY}\times ILI\right)}_{t-i}+s\left(t, df=3\times 6\right)+s\left({AH}_t, df=3\right)\end{array}\right. $$

*Y*_*t*_ denotes the number of P&I deaths in Shanghai at week *t*, which was assumed to follow a quasi-Poisson distribution with expected mean μ_t_ and over-dispersion parameter *φ*; *H1*, *H3*, *BV*, and *BY* denote the first proxy, i.e. weekly positive numbers of lab-confirmed influenza, for influenza A (H1N1), A (H3N2), B (Victoria) and B (Yamagata) viruses at week *t − i*; *i* denotes the lag time between influenza infection and P&I deaths, which varied from 0 to 2 weeks in this study. *s(t, df = 3 × 6)* is the natural cubic spline of time with 3 degrees of freedom per year. *s(AH*_*t*_ *, df = 3)* is the natural cubic spline of absolute humidity to adjust for non-linear association between absolute humidity and deaths. *(LAB*_*H1*_ *)*_*t − i*_, *(LAB*_*H3*_ *)*_*t − i*_, *(LAB*_*BV*_ *)*_*t − i*_, *(LAB*_*BY*_ *)*_*t − i*_ denote the second proxy, i.e. weekly positive proportions of lab-confirmed influenza, for influenza A (H1N1), A (H3N2), B (Victoria) and B (Yamagata) viruses at week *t-i*; and *(LAB*_*H1*_ *)*_*t − i*_ *× ILI*, *(LAB*_*H3*_ *)*_*t − i*_ *× ILI*, *(LAB*_*BV*_ *)*_*t − i*_ *× ILI*, *(LAB*_*BY*_ *)*_*t − i*_ *× ILI* denote the third proxy, i.e. the product of weekly rate of ILI and positive proportion of lab-confirmed influenza, for influenza A (H1N1), A (H3N2), B (Victoria) and B (Yamagata) viruses at week *t-i*. We conducted stratification analysis by classifying the P&I deaths into two age groups of <60 and ≥60 years. Deviance explained by models were considered as the goodness-of-fit index. Autocorrelation in the model residuals were checked by plotting autocorrelation function and partial autocorrelation function of model residuals.

The outcome measurements were excess P&I mortality rates associated with influenza type/subtypes, which was defined as the difference between the fitted number of deaths from the Quasi-Poisson model and the number of baseline deaths. Baseline deaths were obtained by setting the coefficients of influenza subtypes/lineages proxy variables to zero while keeping the other covariates unchanged, assuming there were no influenza subtypes/lineages circulating. Excess mortality rate associated with influenza subtypes/lineages were calculated by dividing the excess number to the general population size or registered population size, respectively. The 95% confidence intervals (CI) for excess mortality were obtained by bootstrapping the residuals 1000 times and refitting the Quasi-Poisson model. Analyses were conducted up to 2 weeks considering the lag effect of influenza on P&I mortality [8].

## Results

### Descriptive statistics

A total of 89,808 specimens were collected and tested for influenza infection during the years 2010–2015, among which 3258 were positive for influenza A (H1N1), 10,579 for influenza A (H3N2), 4125 for influenza B (Victoria) and 4381 for influenza B (Yamagata). The predominant virus was influenza B (Victoria) lineage in the year 2010. The predominant viruses were influenza B (Victoria) lineage and influenza A (H1N1) virus in the year 2011, while the latter shifted to influenza A (H3N2) in the subsequent years 2013 and 2014. The predominant viruses in the year 2015 were influenza A (H3N2) virus and influenza B (Yamagata) lineage (Table 1).

**Table 1:** Annual summary of influenza activity in Shanghai, China from 2010 to 2015

	2010	2011	2012	2013	2014	2015
Total specimens	12,992	10,114	11,828	15,452	19,514	19,908
Positive number						
A (H1N1)	551	760	6	470	853	618
A (H3N2)	802	75	2375	1745	2719	2863
B (Victoria)	1493	801	1774	19	17	21
B (Yamagata)	408	425	73	57	1411	2007
Positive Proportion						
A (H1N1)	4.24	7.51	0.05	3.04	4.37	3.10
A (H3N2)	6.17	0.74	20.08	11.29	13.93	14.38
B (Victoria)	11.49	7.92	15.00	0.12	0.09	0.11
B (Yamagata)	3.14	4.20	0.62	0.37	7.23	10.08
LAB × ILI						
A (H1N1)	30.38	64.06	0.30	23.72	42.81	23.79
A (H3N2)	48.89	4.92	166.55	82.29	133.77	150.13
B (Victoria)	73.49	44.46	104.27	0.84	0.65	0.85
B (Yamagata)	19.64	24.59	4.20	2.85	53.31	79.78
Predominant subtype/lineage	B (Victoria)	A (H1N1); B (Victoria)	A (H3N2); B (Victoria)	A (H3N2)	A (H3N2)	A (H3N2); B (Yamagata)

*LAB × ILI* product of weekly proportion of specimens tested positive for influenza and influenza-like illness consultation rate

**Fig. 1:**
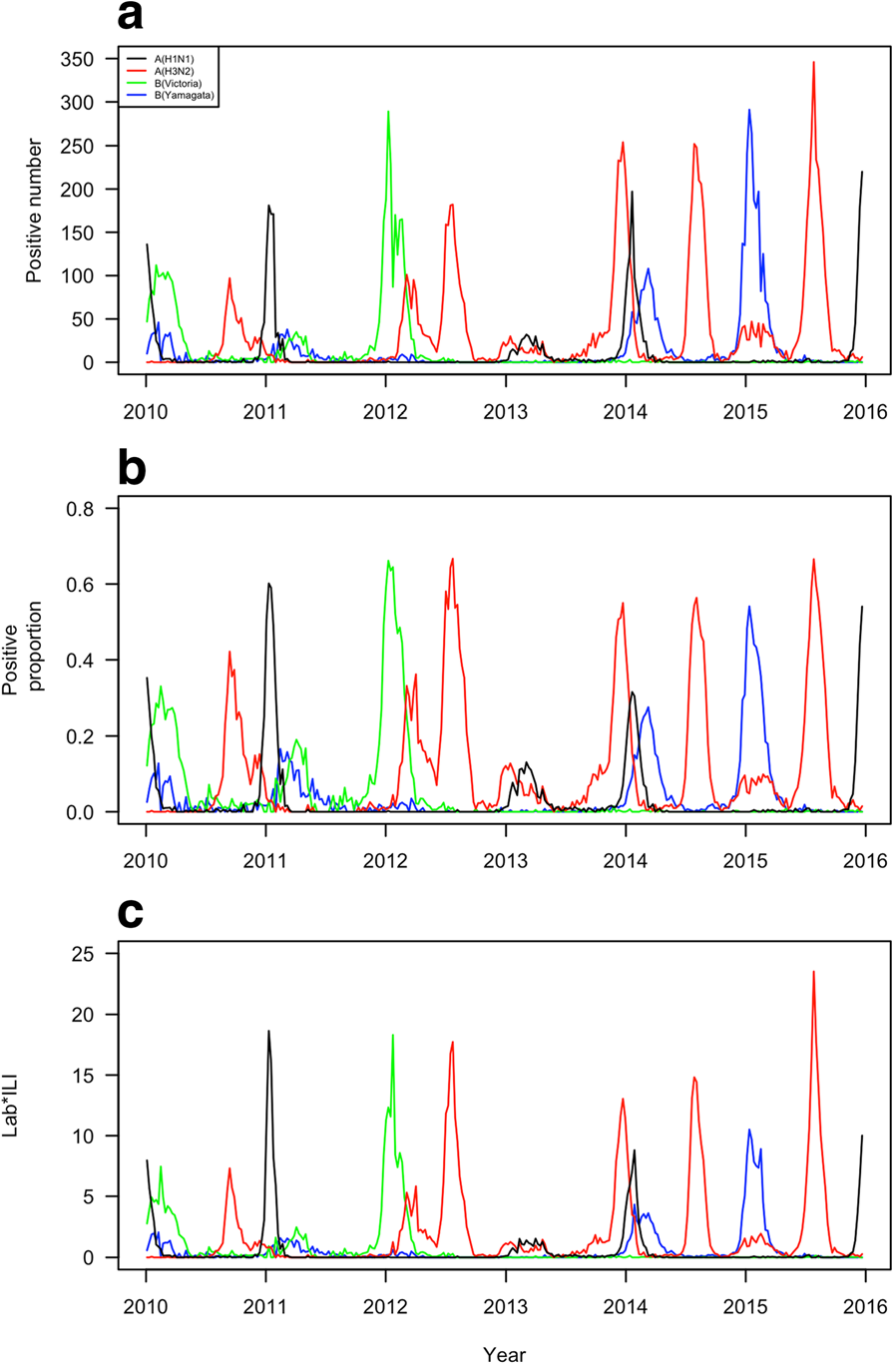
Influenza activity in Shanghai, China from 2010 to 2015. **a** positive number; **b** positive proportion; **c** positive proportion × influenza-like illness

Influenza A (H1N1) virus was active at the beginning of the years 2011, 2013, and 2014 (Fig. 1). Influenza A (H3N2) virus was more active than influenza A (H1N1) virus, which had one or two peaks in all years except 2011 (Fig. 1). In general, influenza A (H3N2) had a higher winter peak than its summer peak. Influenza B (Victoria) virus had only one observable peak in the winter of 2012 while influenza B (Yamagata) virus had two observable peaks in the beginning of 2014 and the beginning of 2015 (Fig. 1).

### Excess mortality associated with influenza by subtypes/lineages

**Fig. 2:**
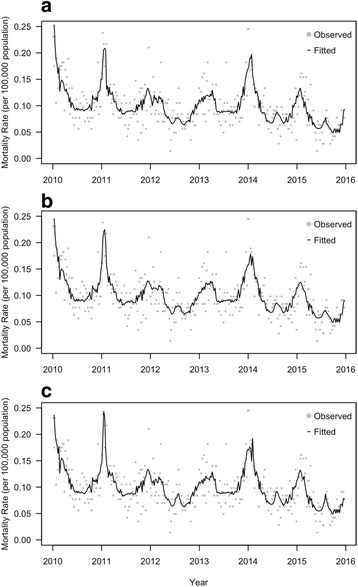
An example of model fit (lag 1 analysis) for deaths coded as pneumonia and influenza in the general population in Shanghai. **a** Proxy: positive number; **b** Proxy: positive proportion; **c** Proxy: positive proportion × influenza-like illness

The quasi-Poisson models generally well fitted the weekly numbers of P&I deaths. An example of the model fit was shown in Fig. 2. Goodness-of-fit statistics and autocorrelation of residuals were presented in Additional file 1. The P&I mortality rate attributable to influenza ranged from 0.14 to 0.31 per 100,000 population. Excess P&I mortality rates were comparable for influenza A subtypes. However, the mortality rate associated with influenza A (H3N2) increased as the lag time between influenza infection and P&I mortality increased. On the other hand, the mortality rate associated with influenza A (H1N1) generally decreased as the lag time increased (Table 2). Compared with influenza A viruses, influenza B viruses showed more moderate mortality burden. One interesting result was that the rate for influenza B (Yamagata) lineage was much higher than that for influenza B (Victoria) lineage (Table 2). In fact, influenza B (Victoria) lineage did not contribute to the estimated excess P&I mortality (*P* = 0.206) while influenza B (Yamagata) lineage was statistically significant (*P* = 0.044).

**Table 2:** Excess pneumonia and influenza mortality rate associated with influenza subtypes/lineages using different influenza proxies in Shanghai general population, 2010–2015

		Excess P&I mortality rate per 100,000 population					
		Lag 0		Lag 1		Lag 2	
		ER	95% CI	ER	95% CI	ER	95% CI
Proxy: positive number							
	A (H1N1)	0.10	(0.06, 0.15)	0.12	(0.07, 0.16)	0.10	(0.06, 0.15)
	A (H3N2)	0.05	(−0.02, 0.11)	0.10	(0.04, 0.17)	0.12	(0.05, 0.18)
	B (Victoria)	−0.05	(−0.12, 0.02)	−0.05	(−0.12, 0.02)	−0.04	(−0.11, 0.03)
	B (Yamagata)	0.04	(−0.02, 0.09)	0.06	(0.01, 0.11)	0.09	(0.04, 0.14)
	Influenza	0.14	(−0.01, 0.27)	0.22	(0.10, 0.34)	0.26	(0.14, 0.37)
Proxy: positive proportion							
	A (H1N1)	0.14	(0.08, 0.19)	0.14	(0.09, 0.19)	0.12	(0.07, 0.17)
	A (H3N2)	0.07	(−0.01, 0.16)	0.12	(0.04, 0.20)	0.14	(0.07, 0.22)
	B (Victoria)	−0.01	(−0.10, 0.06)	−0.03	(−0.10, 0.05)	−0.05	(−0.13, 0.02)
	B (Yamagata)	0.07	(0.01, 0.15)	0.08	(0.01, 0.14)	0.11	(0.05, 0.17)
	Influenza	0.26	(0.08, 0.43)	0.30	(0.14, 0.45)	0.31	(0.17, 0.44)
Proxy: LAB × ILI							
	A (H1N1)	0.10	(0.06, 0.14)	0.09	(0.05, 0.12)	0.08	(0.04, 0.11)
	A (H3N2)	0.07	(0.01, 0.13)	0.11	(0.05, 0.17)	0.12	(0.06, 0.18)
	B (Victoria)	−0.03	(−0.09, 0.04)	−0.04	(−0.10, 0.02)	−0.07	(−0.13, −0.01)
	B (Yamagata)	0.07	(0.01, 0.12)	0.07	(0.02, 0.13)	0.09	(0.04, 0.14)
	Influenza	0.21	(0.09, 0.32)	0.23	(0.11, 0.33)	0.22	(0.11, 0.32)

*P&I* pneumonia and influenza, *ER* Excess rate, *LAB × ILI* product of weekly proportion of specimens tested positive for influenza and influenza-like illness consultation rate, *CI* confidence interval

### Excess mortality for three influenza proxies

The influenza-associated P&I mortality rates for the weekly positive numbers/proportions and LAB × ILI were 0.22, 0.30, and 0.23 per 100,000 persons, respectively at lag 1 week. The excess mortality rate using weekly positive numbers was 4.3% lower than that for LAB × ILI, while the excess mortality rate for the weekly positive proportions was 0.05–0.09 higher than that for LAB × ILI (Table 2).

### Excess mortality for registered population

Excess P&I mortality rate associated with influenza using the weekly positive numbers/proportions and LAB × ILI were 0.34, 0.48, and 0.36 per 100,000 persons, respectively (Table 3). Compared with the excess mortality rate for the general population, the excess mortality rate for the registered Shanghai residents was 36.1% higher. The severity order for influenza subtypes/lineages was likely to be influenza A (H3N2) virus, influenza A (H1N1) virus, and influenza B (Yamagata) lineage. Age-stratified analysis revealed that influenza-associated P&I mortality rates were more than 10-fold higher in the elderly over 60 years of age than whose younger than 60 years of age (Table 4).

**Table 3:** Excess pneumonia and influenza mortality rate associated with influenza subtypes/lineages using different influenza proxies in Shanghai registered residents, 2010–2015

		Excess P&I mortality rate per 100,000 population					
		Lag 0		Lag 1		Lag 2	
		ER	95% CI	ER	95% CI	ER	95% CI
Proxy: positive number							
	A (H1N1)	0.14	(0.06, 0.22)	0.16	(0.09, 0.24)	0.14	(0.08, 0.22)
	A (H3N2)	0.09	(−0.02, 0.20)	0.17	(0.07, 0.28)	0.21	(0.10, 0.31)
	B (Victoria)	−0.08	(−0.19, 0.04)	−0.07	(−0.19, 0.03)	−0.05	(−0.16, 0.05)
	B (Yamagata)	0.06	(−0.03, 0.14)	0.09	(0.00, 0.17)	0.12	(0.04, 0.20)
	Influenza	0.20	(−0.02, 0.41)	0.34	(0.12, 0.53)	0.41	(0.23, 0.59)
Proxy: positive proportion							
	A (H1N1)	0.18	(0.09, 0.26)	0.19	(0.11, 0.27)	0.18	(0.10, 0.25)
	A (H3N2)	0.13	(0.00, 0.27)	0.21	(0.08, 0.33)	0.25	(0.13, 0.36)
	B (Victoria)	−0.01	(−0.15, 0.11)	−0.03	(−0.16, 0.08)	−0.08	(−0.20, 0.04)
	B (Yamagata)	0.11	(0.01, 0.23)	0.12	(0.02, 0.22)	0.16	(0.06, 0.25)
	Influenza	0.40	(0.12, 0.67)	0.48	(0.24, 0.71)	0.50	(0.27, 0.70)
Proxy: LAB × ILI							
	A (H1N1)	0.13	(0.06, 0.19)	0.12	(0.06, 0.18)	0.11	(0.05, 0.17)
	A (H3N2)	0.13	(0.03, 0.23)	0.19	(0.09, 0.28)	0.21	(0.12, 0.30)
	B (Victoria)	−0.03	(−0.13, 0.06)	−0.06	(−0.15, 0.04)	−0.10	(−0.20, −0.01)
	B (Yamagata)	0.10	(0.01, 0.19)	0.11	(0.02, 0.20)	0.14	(0.05, 0.22)
	Influenza	0.31	(0.12, 0.51)	0.36	(0.17, 0.53)	0.35	(0.19, 0.51)

*P&I* pneumonia and influenza, *ER* excess rate, *LAB × ILI* product of weekly proportion of specimens tested positive for influenza and influenza-like illness consultation rate, *CI* confidence interval

**Table 4:** Excess pneumonia and influenza mortality rate associated with influenza subtypes/lineages using different influenza proxies in registered Shanghai residents in 2010–2015, stratified by age

		Excess P&I mortality rate per 100,000 population											
		<60 years of age						≥60 years of age					
		Lag 0		Lag 1		Lag 2		Lag 0		Lag 1		Lag 2	
		ER	95% CI	ER	95% CI	ER	95% CI	ER	95% CI	ER	95% CI	ER	95% CI
Proxy: positive number													
	A (H1N1)	0.04	(0.01, 0.06)	0.04	(0.01, 0.06)	0.03	(0, 0.05)	0.4	(0.12, 0.69)	0.5	(0.24, 0.76)	0.46	(0.22, 0.75)
	A (H3N2)	0.01	(−0.03, 0.04)	0.01	(−0.02, 0.05)	0.02	(−0.01, 0.05)	0.32	(−0.09, 0.71)	0.61	(0.24, 0.98)	0.72	(0.32, 1.08)
	B (Victoria)	−0.01	(−0.06, 0.03)	−0.01	(−0.06, 0.02)	0	(−0.04, 0.03)	−0.26	(−0.69, 0.14)	−0.25	(−0.65, 0.11)	−0.18	(−0.58, 0.19)
	B (Yamagata)	0.01	(−0.03, 0.03)	0	(−0.04, 0.03)	0	(−0.03, 0.03)	0.19	(−0.12, 0.5)	0.32	(0.01, 0.63)	0.45	(0.16, 0.73)
	Influenza	0.04	(−0.04, 0.1)	0.04	(−0.04, 0.1)	0.05	(−0.02, 0.11)	0.65	(−0.13, 1.42)	1.15	(0.42, 1.85)	1.41	(0.75, 2.09)
Proxy: positive proportion													
	A (H1N1)	0.04	(0.01, 0.06)	0.04	(0.01, 0.06)	0.03	(0, 0.05)	0.57	(0.26, 0.86)	0.63	(0.34, 0.92)	0.58	(0.31, 0.85)
	A (H3N2)	0.01	(−0.04, 0.05)	0.01	(−0.03, 0.05)	0.03	(−0.02, 0.06)	0.46	(−0.03, 0.96)	0.74	(0.28, 1.18)	0.85	(0.46, 1.3)
	B (Victoria)	0	(−0.05, 0.04)	0	(−0.05, 0.04)	0.01	(−0.03, 0.05)	−0.04	(−0.56, 0.4)	−0.12	(−0.57, 0.32)	−0.31	(−0.78, 0.11)
	B (Yamagata)	0	(−0.04, 0.04)	−0.01	(−0.04, 0.03)	0.01	(−0.03, 0.04)	0.41	(0.02, 0.82)	0.47	(0.09, 0.87)	0.6	(0.24, 0.95)
	Influenza	0.05	(−0.06, 0.13)	0.05	(−0.06, 0.13)	0.07	(−0.01, 0.13)	1.37	(0.31, 2.35)	1.67	(0.78, 2.51)	1.68	(0.84, 2.51)
Proxy: LAB × ILI													
	A (H1N1)	0.03	(0, 0.05)	0.03	(0.01, 0.05)	0.02	(0, 0.04)	0.41	(0.17, 0.63)	0.39	(0.16, 0.59)	0.36	(0.15, 0.56)
	A (H3N2)	0.01	(−0.03, 0.04)	0.01	(−0.02, 0.04)	0.03	(−0.01, 0.05)	0.46	(0.08, 0.82)	0.66	(0.31, 0.98)	0.72	(0.39, 1.05)
	B (Victoria)	−0.01	(−0.05, 0.03)	0	(−0.04, 0.03)	0.01	(−0.03, 0.04)	−0.11	(−0.49, 0.25)	−0.21	(−0.59, 0.13)	−0.4	(−0.79, −0.06)
	B (Yamagata)	0.01	(−0.03, 0.03)	0	(−0.03, 0.03)	0.01	(−0.03, 0.03)	0.35	(0.01, 0.67)	0.4	(0.07, 0.7)	0.5	(0.2, 0.78)
	Influenza	0.03	(−0.04, 0.09)	0.04	(−0.03, 0.09)	0.06	(−0.01, 0.1)	1.08	(0.4, 1.76)	1.22	(0.57, 1.81)	1.17	(0.55, 1.76)

*P&I* pneumonia and influenza, *ER* excess rate, *LAB × ILI* product of weekly proportion of specimens tested positive for influenza and influenza-like illness consultation rate, *CI* confidence interval

## Discussion

Our estimate for excess P&I mortality rate associated with influenza (0.23 per 100,000 persons) in Shanghai was lower than estimates from other countries, including Australia, New Zealand, Italy, and Singapore [25–28]. In China, our estimate for Shanghai was comparable with that for Hefei city [23], while lower than that for Guangzhou [16], Dalian, Qingdao, and Zhaoyuan cities [4]. In this study, the severity order for influenza subtypes/lineages was likely to be influenza A (H3N2) virus, influenza A (H1N1) virus, and influenza B (Yamagata) virus using LAB × ILI as the proxy, which was the same to that of German and Italy [29, 30]. The excess P&I mortality rate associated with influenza A (H3N2) in this study was 10.5% and 0.7% of that for Italy and German [27, 30], while comparable with that for Hefei [23]. Differences in the quality of the viral surveillance and death surveillance, as well as socio-economic levels, healthcare systems, population immunity and meteorological conditions, may interact together explain the difference of disease burden estimates in China and other countries [31].

One of our most interesting findings was that excess P&I mortality rate associated with influenza B were predominantly attributable to influenza B (Yamagata) virus, while no significant relationships between the other lineage influenza B (Victoria) and excess mortality were found [32, 33]. The WHO has recommended the inclusion of B Victoria lineage for seasons from 2009 to 12 while recommended influenza B Yamagata lineage for seasons from 2012 to 16 in the trivalent inactivated influenza vaccine for Northern Hemisphere [34]. The influenza B lineages in the vaccine composition well matched the circulating influenza B lineages in Shanghai. However, as influenza vaccine is not included in the national immunization program, the coverage of influenza vaccine was very low in China, including Shanghai. The encourage of influenza vaccine uptake in Shanghai residents may help to reduce the burden of influenza, especially when influenza B (Yamagata) lineage attacks.

Another interesting finding was that the performances of different influenza proxies were generally similar with moderate differences. The excess mortality rate fitted using weekly positive proportions of laboratory-confirmed influenza was 0.05–0.12 and 0.05–0.09 higher than those fitted using weekly positive numbers of laboratory-confirmed influenza and LAB × ILI, respectively, while the latter two results were comparable. To our knowledge, LAB × ILI may be more closely related to influenza incidence than positive numbers/proportions of laboratory-confirmed influenza [35]. Excess mortality rate estimated using the positive numbers/proportions of laboratory-confirmed influenza tend to be under-estimated because respiratory samples are often selected for viral testing on the basis of severity of ILI symptoms [8]. According to a previous study, the performance of positive numbers of laboratory-confirmed influenza has a slightly lower bias than the positive proportions of laboratory-confirmed influenza [35]. In general, the Quasi-Poisson models using different influenza proxies provided similar estimates for the excess P&I mortality rate associated with influenza. Further studies are needed to fully compare the performance of these three influenza activity proxies.

The influenza-associated P&I excess mortality rate for the registered Shanghai population was higher than that for the general population in Shanghai. Possible reasons are: (1) over 80% of temporary migrants in Shanghai were constituted of young and middle-aged labor force while seniors over 60 years old only accounted for 3%, who were the high-risk group for influenza [36]. However, the proportion of seniors among Shanghai registered population has exceeded 20% by the year 2009 [37]; (2) many temporary migrants in Shanghai were generally not willing to go to hospitals because they could not afford healthcare, even if they did catch influenza; and (3) a proportion of the temporary migrants in Shanghai might go back to their places of origin when death was approaching because of Chinese tradition, which in turn led to under-reporting of the number of deaths. These findings have important implications for Shanghai’s strategies to prevent and control influenza. First, there may be a need to strengthen vaccination in the elderly population and children of the registered Shanghai residents. Second, further studies need to be done to investigate the influenza-associated excess mortality in the age groups of 0–4 and ≥65 years old. Third, influenza surveillance among the “floating population” should be strengthened to reflect the actual disease burden attributable to influenza.

Our study used statistical modeling methods to estimate he disease burden on influenza with adjustment for environmental confounders of absolute humidity. Several experimental studies have demonstrated that temperature and humidity directly modulated survival and transmission of influenza virus [24, 38]. A confounder was a common cause of exposure and outcome, thus humidity in our study was a confounder because it was associated with influenza virus survival and human deaths. Humidity was also likely to play a role in the causal pathway for influenza transmission, and the adjustment of humidity in this case may underestimate the disease burden of influenza. However, it was hard to quantify to what extent the humidity played a role in influenza virus survival and transmission respectively. We therefore adjusted the absolute humidity as a confounder as previous studies did [23, 39]. We did not add temperature in our model to avoid collinearity problem as the correlation between temperature and absolute humidity was over 0.95 in Shanghai.

Our study has several potential caveats. First, this study only examined P&I mortality due to data access, without mortality from all-cause death or respiratory and circulatory system disease (R&C) death, thus not featuring a complete and comprehensive assessment of the impact of influenza on death. Second, the mortality data in this study was retrieved from the SCDC, any omission, misstatement might lead to deviations to result. Third, in addition to the confounding factors adjusted in this study (short-term trends, long-term trends and humidity), there might be other unmeasured factors that would lead to bias.

## Conclusions

Influenza A (H3N2) appeared to be the most active influenza subtype and showed clear winter peak, with a less intense summer peak. Compared with influenza A viruses, influenza B viruses showed more moderate mortality burden contributed by Yamagata lineage. In general, quasi-Poisson models using different influenza proxies provided similar estimates for the excess P&I mortality rate associated with influenza. Compared with the excess mortality rate for the general population, the excess mortality rate for the registered Shanghai residents was higher. Our findings provide scientific evidence for policy-maker in making preventive and control strategies for influenza epidemics.

## Additional file

Additional file 1: Goodness-of-fit of models. **Table S1.** Deviance explained for models using three different proxy variables for influenza activity; **Figure S1.** Plots of residual autocorrelation function and partial autocorrelation function of quasi-Poisson models at lag 1 using proxies A) number; B) proportion; C) product of weekly proportion of specimens tested positive for influenza and influenza-like illness consultation rate. (PDF 513 kb)
