# Supplementary material for: Excess pneumonia and influenza mortality attributable to seasonal influenza in subtropical Shanghai, China
Source: BMC Infect Dis. 2017 Dec 7;17:756. doi: 10.1186/s12879-017-2863-1 (PMC5719671; doi:10.1186/s12879-017-2863-1)

Additional file *Goodness-of-fit of models*

Table S1. Deviance explained by models using three different proxy variables for influenza activity

	Deviance explained (%)		
	Lag 0	Lag 1	Lag 2
General population			
positive number	0.512	0.524	0.526
positive proportion	0.519	0.523	0.531
LAB×ILI	0.527	0.526	0.535
Registered population			
positive number	0.493	0.510	0.515
positive proportion	0.498	0.508	0.519
LAB×ILI	0.504	0.512	0.526

LAB×ILI: product of weekly proportion of specimens tested positive for influenza and influenza-like illness consultation rate.

Figure S1. Plots of residual autocorrelation function and partial autocorrelation function of quasi-Poisson models at lag 1 using proxies A) number; B) proportion; C) product of weekly proportion of specimens tested positive for influenza and influenza-like illness consultation rate.

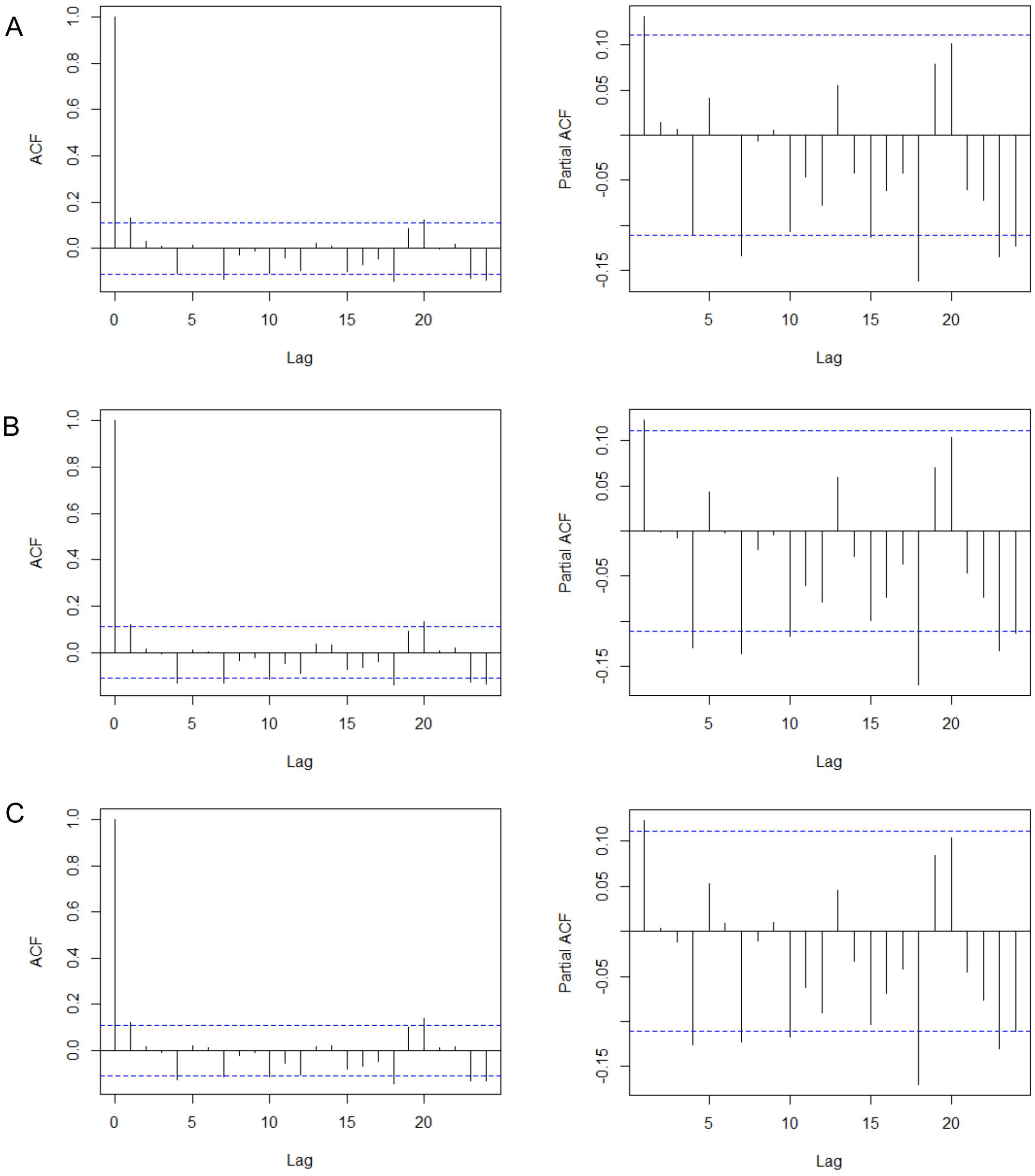

Supplement: Additional file 1: — Goodness-of-fit of models. Table S1. Deviance explained for models using three different proxy variables for influenza activity; Figure S1. Plots of residual autocorrelation function and partial autocorrelation function of quasi-Poisson models at lag 1 using proxies A) number; B) proportion; C) product of weekly proportion of specimens tested positive for influenza and influenza-like illness consultation rate. (PDF 513 kb) [file 12879_2017_2863_MOESM1_ESM.pdf]
